# SPX family response to low phosphorus stress and the involvement of *ZmSPX1* in phosphorus homeostasis in maize

**DOI:** 10.3389/fpls.2024.1385977

**Published:** 2024-07-08

**Authors:** Bowen Luo, Javed Hussain Sahito, Haiying Zhang, Jin Zhao, Guohui Yang, Wei Wang, Jianyong Guo, Shuhao Zhang, Peng Ma, Zhi Nie, Xiao Zhang, Dan Liu, Ling Wu, Duojiang Gao, Shiqiang Gao, Shunzong Su, Zeeshan Ghulam Nabi Gishkori, Shibin Gao

**Affiliations:** ^1^ State Key Laboratory of Crop Gene Exploration and Utilization in Southwest China, Chengdu, Sichuan, China; ^2^ Maize Research Institute, Sichuan Agricultural University, Chengdu, Sichuan, China; ^3^ Key Laboratory of Biology and Genetic Improvement of Maize in Southwest Region, Ministry of Agriculture, Chengdu, Sichuan, China; ^4^ National Key Laboratory of Wheat and Maize Crop Science, College of Agronomy, Henen Agricultural University, Zhengzhou, China; ^5^ Maize Research Institute, Mianyang Academy of Agricultural Sciences, Mianyang, Sichuan, China; ^6^ Sichuan Academy of Agricultural Sciences, Biotechnology and Nuclear Technology Research Institute, Chengdu, Sichuan, China; ^7^ College of Resources, Sichuan Agricultural University, Chengdu, Sichuan, China; ^8^ Institute of Biotechnology, College of Agriculture and Biotechnology, Zhejiang University, Hangzhou, China

**Keywords:** maize, low-Pi stress, *SPX* gene family, *PHRS*, candidate gene association analysis

## Abstract

Phosphorus (P) is a crucial macronutrient for plant growth and development, and low-Pi stress poses a significant limitation to maize production. While the role of the SPX domain in encoding proteins involved in phosphate (Pi) homeostasis and signaling transduction has been extensively studied in other model plants, the molecular and functional characteristics of the *SPX* gene family members in maize remain largely unexplored. In this study, we identified six *SPX* members, and the phylogenetic analysis of *ZmSPX*s revealed a close relationship with *SPX* genes in rice. The promoter regions of *ZmSPX*s were abundant in biotic and abiotic stress-related elements, particularly associated with various hormone signaling pathways, indicating potential intersections between Pi signaling and hormone signaling pathways. Additionally, *ZmSPX*s displayed tissue-specific expression patterns, with significant and differential induction in anthers and roots, and were localized to the nucleus and cytoplasm. The interaction between *ZmSPX*s and *ZmPHR*s was established via yeast two-hybrid assays. Furthermore, overexpression of *ZmSPX1* enhanced root sensitivity to Pi deficiency and high-Pi conditions in *Arabidopsis thaliana*. Phenotypic identification of the maize transgenic lines demonstrated the negative regulatory effect on the P concentration of stems and leaves as well as yield. Notably, polymorphic sites including 34 single-nucleotide polymorphisms (SNPs) and seven insertions/deletions (InDels) in *ZmSPX1* were significantly associated with 16 traits of low-Pi tolerance index. Furthermore, significant sites were classified into five haplotypes, and haplotype5 can enhance biomass production by promoting root development. Taken together, our results suggested that *ZmSPX* family members possibly play a pivotal role in Pi stress signaling in plants by interacting with *ZmPHR*s. Significantly, *ZmSPX1* was involved in the Pi-deficiency response verified in transgenic *Arabidopsis* and can affect the Pi concentration of maize tissues and yield. This work lays the groundwork for deeper exploration of the maize *SPX* family and could inform the development of maize varieties with improved Pi efficiency.

## 1 Introduction

Maize (*Zea mays* L.) stands as a pivotal crop globally, not only as a source of food and high-quality feed but also for industrial applications (Feng et al., 2022; Jiao et al., 2023). P is an essential macronutrient for plant growth and development and plays a vital role either directly or indirectly in many physiological and biochemical processes, such as photosynthesis, respiration, signal transduction, and metabolic processes (Luo et al., 2019). Plants primarily acquire P in the inorganic form of Pi from the soil to support their growth and yield (Kumar et al., 2019). The low availability of Pi in soils is a significant limiting factor for plant growth and yield, posing a significant challenge (Balyan et al., 2016). Consequently, the growing need for increased food production and higher crop yields is expected to lead to a rise in the demand for Pi inputs in cropland. The global application of Pi fertilizer on croplands has increased several times from 1961 to 2013 and already surpassed the estimated planetary boundary (Zou et al., 2022). Excessive fertilizer application has led to a myriad of environmental and ecological issues, including water eutrophication.

In response to low-Pi stress, plants have developed a series of adaptive mechanisms, primarily manifested through changes in root morphological structure, physiological and biochemical regulation, and the expression of Pi starvation-inducible genes. Specifically, under low-Pi stress, alterations in root morphology and configuration were characterized by the inhibition of primary roots, accompanied by an increase in the length and quantity of lateral roots and root hairs (Raghothama, 1999; Lynch, 2011). Additionally, the root radius decreases, resulting in a higher root–shoot ratio and a shallower root system. These morphological changes facilitate plants in expanding the contact area between roots and the superficial soil (Raghothama, 1999; Lynch, 2011; Luo et al., 2023). Furthermore, low-Pi stress triggers modifications in plant enzyme system activity, further contributing to the plant’s adaptation to Pi scarcity. For example, Pi deficiency inhibits the activity of ATP synthase, which subsequently impacts ATP and NADPH production, thereby reducing the photosynthetic rate, carbon fixation, and overall plant growth (Carstensen et al., 2018; Garcia et al., 2023; Iqbal et al., 2023; Saengwilai et al., 2023). Enhancing the activities of sulfur lipid and glycolipid synthases, as well as phospholipid hydrolases, contributes to altering the composition of the lipid membrane. Moreover, low-Pi stress can elevate the activity of defense-related enzymes, enabling plants to better adapt to the low-Pi environment (Shimojima, 2011; Liang et al., 2014; Chen et al., 2015). Additionally, the increase in root exudates represents another strategy for plants to cope with low-Pi stress. This includes the secretion of protons, organic acids, and acid phosphatase, which serve to acidify the soil surrounding the roots and release inorganic Pi for plant uptake (Lynch et al., 2005; Shen et al., 2005; Zhang et al., 2010). When encountering Pi deficiency, plants also maintain Pi homeostasis through a series of molecular reactions. For instance, the phosphate starvation response (PSR) pathway involves a MYB transcription factor known as *PHR1*, which serves as a key regulator in PSR. It governs transcription and takes part in physiological and biochemical adaptations (Zhang et al., 2014). PHR1 is capable of directly binding to the *cis*-elements P1BS (PHR binding sequence, GNATATNC), which were found in the promoter regions of several PSR genes, including Phosphate 1 (*PHO1*), phosphate transporter traffic facilitator 1 (*PHF1*), phosphate transporters (PTs), induced by phosphate starvation (*IPS1*), miRNA399, and miRNA827 (Rubio et al., 2001; Puga et al., 2017). As Pi sensors, SPX proteins can interact with AtPHR1 or OsPHR2, and under normal conditions, they inhibit their transcriptional activity. This interaction between SPX proteins and PHR transcription factors serves to prevent the toxicity resulting from excessive Pi accumulation (Wang et al., 2014). Notably, the SPX proteins themselves do not directly sense Pi but instead sense soluble inositol polyphosphates (InsPs) with a high affinity (Wild et al., 2016). Recently, the crystal structure of the SPX domain revealed the basic surface for InsP6, and biochemical studies have shown that InsP7 stimulated the interaction between *OsSPX4* and *OsPHR2* with higher binding affinity than InsP6 (Jung et al., 2018). Among these, InsP8 acts as an intracellular Pi signaling substance, regulating Pi balance by modulating the interaction between *AtSPX1* and *AtPHR1* (Dong et al., 2019). Consequently, *SPX* genes play a crucial role in Pi signaling pathways and homeostasis in plants (Li et al., 2021).

The SPX proteins, namely, SYG1 (suppressor of yeast gpa1), Pho81 (CDK inhibitor in the yeast PHO pathway), and XPR1 (xenotropic and polytropic retrovirus receptor), can be classified into four subfamilies based on the presence of structural characteristics: class 1 only contained SPX domain, and the other three have (SPX-MFS, SPX-EXS, and SPX-RING) domains (Chiou and Lin, 2011; Yang et al., 2017; Yue et al., 2017). Recently, two additional classes of SPX proteins, namely, SPX-SLC and SPX-VTC, had been characterized in algae, and they were involved in Pi synthesis and transportation in vacuoles. However, these classes appear to have been lost throughout the evolution of plants, with the type of Pi storage changing from polyphosphates in algae to Pi in the later-diverging *streptophytes* (Wang et al., 2021a). It seems that the SPX domain has some extra domains that may have been lost during the evolution of SPX proteins and have not been comprehensively identified yet (Kopriva and Chu, 2018; Nezamivand-Chegini et al., 2021). *SPX* gene family members have been studied for their significant roles in Pi signaling and homeostasis in various plant species, including four *SPX*s in *Arabidopsis thaliana* and six *SPX*s in rice (Secco et al., 2012). In *A. thaliana*, *AtSPX1* and *AtSPX3* played a positive role in plant adaptation to Pi starvation. Additionally, *AtSPX1* and *AtSPX3* demonstrated redundancy, and *AtSPX3* may negatively regulate the expression of *AtSPX1* (Duan et al., 2008). Moreover, under normal-Pi conditions, *AtSPX4* acted as a negative regulator of PSR gene expression in shoots, influencing both PHR1-dependent and PHR1-independent Pi starvation responses. Disruption of the *AtSPX4* function led to excessive Pi accumulation in the shoots (Osorio et al., 2019). In rice, *OsSPX3*/*5* negatively regulated the transport of Pi from roots to shoots. *OsSPX4* interacted with *OsPHR2* in the cytoplasm, inhibiting Pi signaling. Meanwhile, *OsSPX6* was upregulated under low-Pi stress, leading to the activation of *OsPHR2* (Lv et al., 2014; Zhong et al., 2018). Furthermore, OsSPX4 was involved in the regulation pathway of nitrogen and Pi signals, and ubiquitinated OsSPX4 facilitated the activation of Pi signaling by nitrate, coordinating the utilization of nitrogen and Pi (Hu et al., 2019). Moreover, *SPX* genes have been reported via bioinformatics analysis in diverse species (Yao et al., 2014b; Du et al., 2017; Kumar et al., 2019; Xiao et al., 2021) because of their possible involvement in a number of physiological and molecular processes. However, despite these advancements, the specific functions of these *SPX* gene family members have remained unknown in maize. In our previous study, SPX proteins carrying only the SPX domain were predicted under low-Pi conditions in maize (Nie et al., 2021). Therefore, in the present study, we conducted a bioinformatics analysis to investigate the relationship of SPX proteins containing only the SPX domain across various species. Additionally, we explored the expression patterns of these *SPX* genes in maize, examined their interaction with PHRs, and elucidated the function of *ZmSPX1* through transgenic experiments and candidate gene association analysis in maize.

Taken together, our findings suggested the involvement of *ZmSPX*s in transcription regulations during Pi starvation and found that *ZmSPX1* was involved in response to low-Pi conditions and provide a solid foundation for a deeper understanding of the molecular mechanism of genes and improving P use efficiency (PUE) in maize and will promote future studies on this important gene family in plants.

## 2 Materials and methods

### 2.1 Plant material and growth conditions

Using the CRISPR/Cas9 (Clustered regularly interspaced short palindromic repeats/CRISPR-associated protein 9) system, the *ZmSPX1* gene was knocked out in the maize inbred line KN5585 by the *Agrobacterium tumefaciens*-mediated transformation method. The mutation site was detected using ZmSPX1-KO-F and R primers (**Supplementary Figure S1**; **Supplementary Table S1**). The construction method for maize overexpressing lines utilizes the homologous recombination method to introduce the coding sequence (CDS) of the *ZmSPX1* gene into the vector pCAMBIA3301. Subsequently, *A. tumefaciens*-mediated transformation of the maize recipient line KN5585 was employed to generate transgenic plants. The results for the detection of overexpression and the specific primers used for detection are provided in **Supplementary Figure S2** and **Supplementary Table S1**, respectively. Seeds of 178 (low-Pi tolerant maize inbred line), *A. thaliana* (Columbia), and tobacco (*Nicotiana benthamiana*) were provided by the Maize Research Institute of Sichuan Agricultural University. A relatively low-Pi field site in Wenjiang farm (WJ, Chengdu, Sichuan Province, plain region, available Pi 22.8 mg/kg) of Sichuan Agricultural University was selected for the experiments on low-Pi treatment of maize inbred line 178. Each plot received two levels of Pi treatments (low- and normal-Pi conditions), with each treatment replicated thrice. The plots measured 3 m in length with 0.8 m between rows. Prior to reaching the five-leaf stage, plant thinning reduced the number to 14 plants per plot (58,000 plants/ha). In the normal-Pi treatment plots, the following fertilizers (Stanley Agriculture Group Co., Ltd.) were applied: 150 kg of urea, 900 kg of calcium superphosphate, and 350 kg of potassium chloride per hectare before planting; 130 kg of urea per hectare at the six-leaf stage; and 210 kg of urea per hectare as a side dressing prior to tasseling. The low-Pi treatment plots received the same fertilizer combination as the normal-Pi plots, with the exception of the calcium superphosphate. The seeds of *A. thaliana* were planted in nutrient soil and soil sterilized at 121°C for 20 min and cooled for 30 min. Then, seeds were cultured in nutrient soil at 23°C for 12 h of light and darkness until the *A. thaliana* mature.

### 2.2 Bioinformatics identification of *ZmSPX*s in maize

Based on our previous research (Nie et al., 2021), *ZmSPX* homologous genes were identified with high similarity sequence: *ZmSPX1* (GRMZM2G035579), *ZmSPX2* (GRMZM2G171423), *ZmSPX3* (GRMZM2G024705), *ZmSPX4* (GRMZ2G122108), *ZmSPX5* (GRMZM5G828488), *ZmSPX6* (GRMZM2G065989), and *ZmSPX7* (GRMZM2G083655). The *ZmSPX* family members were identified in maize, and the reliability of the *ZmSPX* CDS was predicted using BioXM 2.6 software. The conserved domain of *ZmSPX*s was verified using the National Center for Biotechnology Information (NCBI) search database (Marchler-Bauer et al., 2015). Amino acid sequences query homologous amino acid sequences of *SPX*s in maize, wheat, soybean, rape, rice, and *Arabidopsis* using the BLASTP program from the NCBI (http://www.ncbi.nlm.nih.gov); phylogenetic relationships were aligned using Mega (version 11.0.13) (Kumar et al., 1994). The gene IDs of the *SPX* gene family in other species were acquired from previous research studies (Secco et al., 2012; Yao et al., 2014b; Du et al., 2017; Kumar et al., 2019). Finally, *ZmSPX*s were studied for cloning and functional analyses based on protein comparison and transcriptome sequencing, respectively. The promoter region (2,000 bp) of *ZmSPX*s was utilized to predict the functional region of these genes using the BDGP online database (http://www.fruitfly.org/seq_tools/promoter.html) (**Supplementary Table S2**), and the *cis*-acting elements within the promoter were predicted using the plantCARE web server (http://bioinformatics.psb.ugent.be/webtools/plantcare/html).

### 2.3 Cloning of *ZmSPX* family members in maize

Total RNA was extracted from the leaves and roots of 178 inbred lines following the manufacturer’s protocol of Thermo Fisher Scientific, Life Technologies (TRIzol Reagent^®^; the protocol can be found at https://www.thermofisher.com). The cDNA reverse transcription was performed using the PrimeScript™ II 1st Strand cDNA Synthesis Kit (Takara Bio Inc., Otsu, Japan). The CDS of *ZmSPX*s was then amplified using the cDNA as a template. The polymerase chain reaction (PCR) primers were designed based on the CDS of the B73 reference, targeting the specific sequences of the *ZmSPX* family members using Primer Premier 5 software. The forward and reverse primers are listed in **Supplementary Table S3**. The PCR was performed in a 25-µL volume containing phanta max buffer 12.5 μL (Phanta Max Super-Fidelity DNA Polymerase, Nanjing Vazyme Biotech Co., Ltd., Nanjing, China), phanta max super fidelity 0.5 μL, dNTP 4 μL, cDNA 1 μL, 1 μL of each forward and reverse primer, and 7 μL ddH_2_O. PCR was programmed for 3 min at 95°C followed by 30 cycles of 95°C for 15 s, annealing for 15 s, 72°C for 45 s, extension for 5 min at 72°C, and final 12°C for preservation. Then, PCR products were separated using 1% agarose gel, purified using a DNA purification kit, and cloned into pEASY^®^-Blunt zero Cloning Vector (TransGen Biotech Co., Ltd., Beijing, China) according to the manufacturer’s protocol. Finally, positive clones were selected for sequencing by TsingKe Biological Technology Co., Ltd. (Beijing, China). The coding sequence of *ZmSPX*s was compared with that of the B73 reference inbred line using DNAMAN v6.0 software.

### 2.4 Expression pattern of *ZmSPX* family members under Pi deficiency in different tissues

Different maize tissues were harvested in the silking stage from normal-Pi and low-Pi treatments. Total RNA was extracted from different maize tissues of 178 inbred lines including leaf, stem, anther, cornsilk, root, ear, and ear bract according to the TRIzol Reagent^®^ (Invitrogen, Carlsbad, CA, USA) manufacturer’s instructions, and reverse transcription was performed using PrimeScript™ II 1st Strand cDNA synthesis kit (Takara Bio Inc., Otsu, Japan). Relative quantitative results were calculated by normalization to the reference gene (GAPDH). At least three independent experiments were performed, and each experiment was performed in technical triplicate. All primers used in the qRT-PCR assay were designed in BEACON DESIGNER 7 and are listed in **Supplementary Table S4**. qRT-PCR data were analyzed using the 2^−ΔΔCT^ method.

### 2.5 Subcellular localization of ZmSPX protein

The full-length CDS region was cloned into plant expression vector pCAMBIA2300 for the *ZmSPX*s. The homologous recombination primers with enzyme cutting sites (*Sma*I and *Xba*I) were designed by CEDesignV1.04 software and are shown in **Supplementary Table S5**. The subcellular localization was assayed in tobacco leaves according to the previously reported transit transformation method (Zhou et al., 2018; Sahito et al., 2020a, b). The transformed tobacco epidermal cells were observed using the A1R-si laser confocal microscope (LSCM, Nikon, Tokyo, Japan) to visualize the green fluorescent protein (GFP) fluorescent signals. The experiment was repeated at least three times to ensure consistency of results.

### 2.6 Yeast two-hybrid assays of ZmSPXs and ZmPHRs

The full-length CDSs of *ZmSPX* family members *ZmPHR1* (GRMZM2G006477) and *ZmPHR2* (GRMZM2G162409) were amplified using gene-specific primers from the cDNA samples. The primers were designed using CE design v1.04 and Primer 5.0 to incorporate (*Eco*rR I, *Bam*H I, and *Nde* I) restriction sites, as specified in **Supplementary Table S6**. Phanta Max high-fidelity DNA polymerase (Nanjing Vazyme Biotech Co., Ltd.) was used to amplify the targeted fragments, and then the amplified fragments were inserted into bait (pGBKT7) and prey (pGADT7) vectors. All possible combinations were co-transferred into the Y2HGold yeast-competent cell. pGBKT7-53 and pGBKT7-lam were used as positive and negative controls, respectively. To exclude the possible autoactivation of *ZmSPX* members, a control experiment was carried out by transformation of loaded bait and prey plasmids with empty prey and bait plasmids, respectively. After screening on solid DDO (−Leu/−Trp) medium for 2–4 days at 28°C, selected monoclonals were inoculated in liquid DDO (−Leu/−Trp) medium until OD600 = 0.3–0.5. These cultures were inoculated on QDO (SD/−Ade/−His/−Trp/−Leu/X-α-Gal) medium after 10-fold dilution. Results were observed after 3–5 days of incubation at 30°C.

### 2.7 Overexpression of *ZmSPX1* in *A. thaliana*

Cloning of the full open reading frame (ORF) of *ZmSPX1* into the vector pCAMBIA3300-35s-PROII MCS-bar and subsequent transformation into wild-type (WT) *Arabidopsis* produced several transformants through the floral dip method previously described (Clough and Bent, 1998). The seeds of the T0 generation were harvested and sown in the soil to select positive transgenic seedlings. Two-week-old seedlings of T1 plants were screened by spraying with Basta (1/1,000) solution. After the transgenic plants were harvested, DNA was extracted, and PCR was performed to confirm the positive *ZmSPX1* transgenic lines.

### 2.8 Phenotypic characterization of *ZmSPX1* transgenic lines under low-Pi stress

The seeds of both WT and *ZmSPX1* transgenic lines were sterilized with 75% (v/v) ethanol for 60–90 s and 2% sodium hypochlorite for 8–15 min, followed by several rinses with distilled water. Fifty seeds of both WT and transgenic lines were cultured on 1/2 normal MS agar medium, initially kept at 4°C for 2 days, and subsequently transferred to a greenhouse with a light cycle of 16 hours at 22°C and a dark cycle of 8 hours at 18°C for 7 days. After 7 days, seedlings were transferred to 1/8 normal MS agar medium and subjected to different Pi concentrations: 0 mmol/L (control), low Pi (0.1 mmol/L), normal Pi (1 mmol/L), and high Pi (10 mmol/L). The seedlings were kept in a controlled environment for 2 weeks. Phenotypic observations under different Pi concentrations were recorded after 2 weeks, and their roots were scanned using a WinRHIZO root-scanning method. All *Arabidopsis* seedlings were dried and digested for the detection of P and nitrogen concentration through a chemical continuous flow analyzer.

### 2.9 Sequencing and association analysis of *ZmSPX1* in maize association population

A total of 211 out of 360 inbred lines were screened from the current Southwest China breeding program (Zhang et al., 2016). These lines were used for association analysis of *ZmSPX1* in maize. The genomic DNA was extracted from leaves at the seedling stage according to the cetyltrimethylammonium bromide (CTAB) method (Porebski et al., 1997). The genomic sequence of *ZmSPX1* from the B73 inbred line was utilized as the reference sequence and obtained from the Maize Genomic Database (http://www.maizegdb.org). The targeted fragment was amplified using high-fidelity phanta max enzyme polymerase (Vazyme Biotech Co., Ltd.). Amplified fragments were sequenced by TsingKe Biological Technology Co., Ltd. Then, the targeted sequence of *ZmSPX1* was compared with the reference genomic sequence of the B73 using DNAMAN v6.0 software, and the sequences of 211 maize inbred lines were trimmed neatly using Bio-Edit 7.1 software (Strable and Scanlon, 2009). Single-nucleotide polymorphisms (SNPs) and insertions/deletions (InDels) were identified in all tested inbred lines with a <0.05 minor allele frequency (MAF), and linkage disequilibrium (LD) between two polymorphic sites was generated using Tassel software v2.1 (Bradbury et al., 2007). Association analysis was carried out between SNPs and InDels and 22 phenotypic traits using Tassel software (Bradbury et al., 2007; Luo et al., 2019). The standard mixed linear model (MLM) including a population structure (Q) and kinship matrix (K) was chosen to detect the significant association of SNPs and InDels as described previously (Zhang et al., 2016; Luo et al., 2019). The association of sites was considered significant at *p* < 0.05, and the calculated *p*-values were converted into −log_10_^(^ *^p^* ^value)^.

## 3 Results

### 3.1 Evolutionary tree analysis of *SPX*s in plants

**Figure 1:**
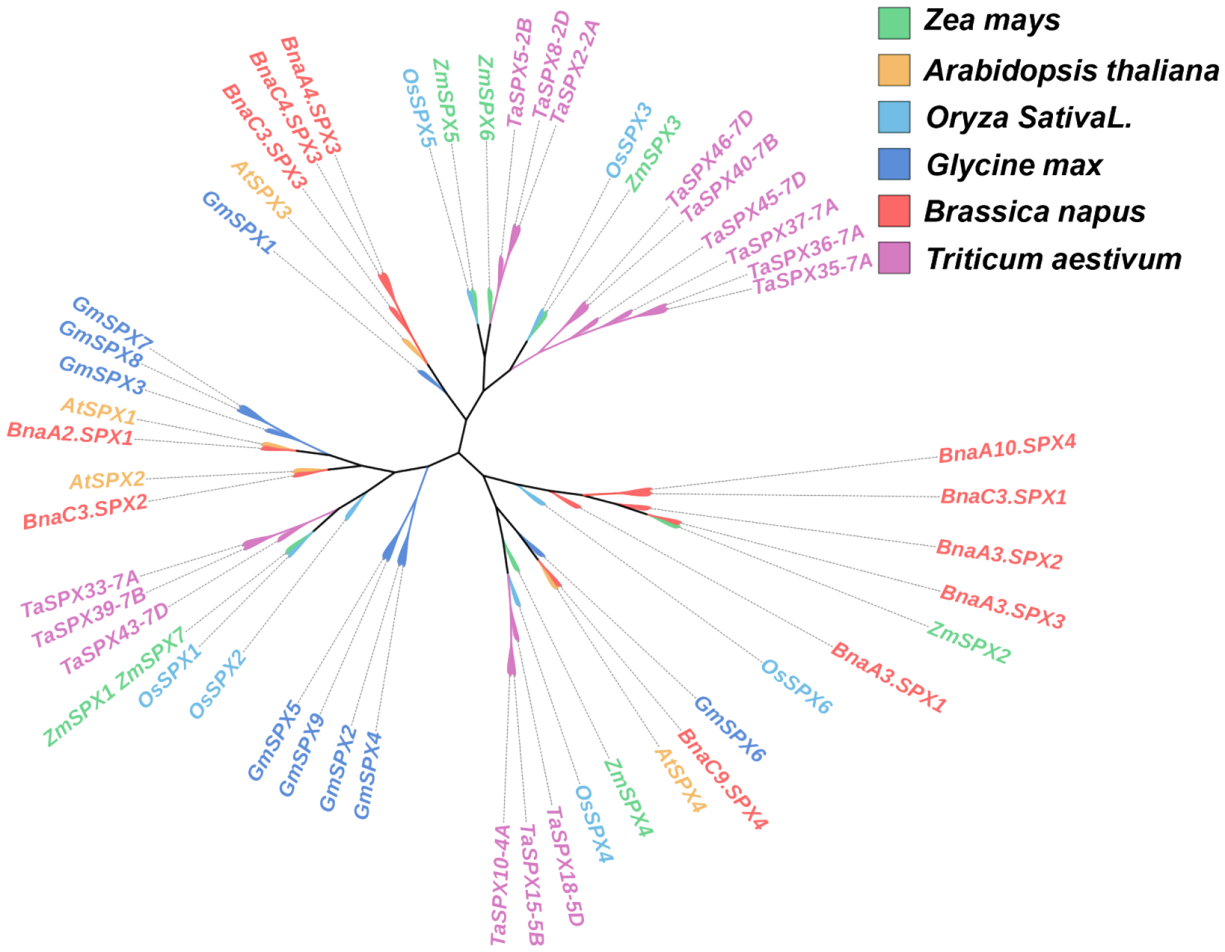
Phylogenetic tree of *SPX*s in plants. The phylogenetic tree was created in the Mega 11 program. In plants, proteins harboring the SPX domain are classified into four families based on the presence of additional domains in their structure, namely, the SPX, SPX-EXS, SPX-MFS, and SPX-RING families. The SPX proteins of this phylogenetic tree carry only the SPX domain.

The *SPX* homologous genes *ZmSPX1* (GRMZM2G03557), *ZmSPX2* (GRMZM2G171423), *ZmSPX3* (GRMZM2G024705), *ZmSPX4* (GRMZM2G122108), *ZmSPX5* (GRMZM5G828488), *ZmSPX6* (GRMZM2G065989), and *ZmSPX7* (GRMZM2G0836555) were predicted in our previous study, and genes were located on different chromosomes (**Supplementary Table S7**). However, in the fourth edition of the maize genome, *ZmSPX1* and *ZmSPX7* were merged into a single gene. Additionally, preliminary transcriptome results indicated that *ZmSPX7* (GRMZM2G0836555) did not respond to low-Pi stress (**Supplementary Table S7**). Hence, in subsequent experiments, the ZmSPX1–6 in the third version of the maize genome were exclusively analyzed. Furthermore, *ZmSPX1*, *ZmSPX2*, *ZmSPX3*, *ZmSPX4*, *ZmSPX5*, and *ZmSPX6* were amplified in maize according to the targeted CDS by PCR. Specifically, the CDS length of *ZmSPX1* was 354 bp, encoding a protein comprising 117 amino acids. Correspondingly, *ZmSPX2*, *ZmSPX3*, *ZmSPX4*, *ZmSPX5*, and *ZmSPX6* have CDS lengths of 846 bp, 687 bp, 996 bp, 765 bp, and 759 bp, encoding proteins of 281, 228, 331, 254, and 252 amino acids, respectively. Simultaneously, *SPX* genes of wheat, soybean, rape, rice, and *Arabidopsis* were also searched for in the database (Secco et al., 2012; Yao et al., 2014b; Du et al., 2017; Kumar et al., 2019). Among them, *Arabidopsis* has four *SPX* genes (*AtSPX1*–*4*), rice has six *SPX* genes (*OsSPX1*–*6*), soybean has nine *SPX* genes (*GmSPX1*–*9*), and rape and wheat have 11 and 15 *SPX* genes, respectively (**Supplementary Table S8**). To deepen our understanding of the evolutionary relationships between these SPX proteins, a phylogenetic tree based on the SPX proteins in these plants was constructed (**Figure 1**). The six *SPX* genes of maize were distributed across different clades. Notably, *ZmSPX5* and *ZmSPX6* displayed the closest evolutionary relationship, clustering within the same clade but occupying distinct branch points (**Figure 1**). Interestingly, the closest evolutionary relationship existed between the *SPX* genes in maize and rice. Specifically, *ZmSPX5* and *OsSPX5*, *ZmSPX3* and *OsSPX3*, and *ZmSPX1* and *OsSPX1* were all located at the same branch point (**Figure 1**). Furthermore, while *ZmSPX4* and *OsSPX4* were positioned at different branch points on the evolutionary tree, they belonged to the same clade and were situated at adjacent branch points (**Figure 1**). Remarkably, *ZmSPX2* showed a close evolutionary relationship with *BnaA3SPX3* (**Figure 1**).

### 3.2 *cis*-Acting element analysis of promoter region of *ZmSPX*s

Generally, the results showed that *ZmSPX* gene family members were associated with abscisic acid response, light reaction, methyl jasmonate (MeJA) reaction, low-temperature response, auxin response, and regulation of zein metabolism *cis*-acting elements (**Supplementary Table S9**). Specifically, *ZmSPX* gene family members also possess unique *cis*-acting regulatory elements. For instance, *ZmSPX1* contains response elements for gibberellin and salicylic acid. The *ZmSPX2* gene responds to light and is also involved in defense and stress responses. *ZmSPX3* is involved in the light responsiveness. *ZmSPX4* contains a conserved DNA module (ATCT-motif) involved in photoreaction. *ZmSPX5* is involved in anaerobic induction. *ZmSPX6* contains DNA-binding protein binding sites and responds to light. It also contains MYB binding sites involved in the regulation of flavonoid biosynthesis genes. Previous studies have demonstrated that *SPX*s can interact with the MYB domain and the CC domain of PHRs (Jia et al., 2023). Additionally, our results also indicated that *ZmSPX* gene family members also contain MYB binding sites or MYB recognition sites (**Supplementary Table S9**). All of these findings suggested that *ZmSPX*s may play a role in the regulation of *ZmPHR*s in association with certain hormones.

### 3.3 Expression pattern of *ZmSPX*s under low- and normal-Pi conditions

**Figure 2:**
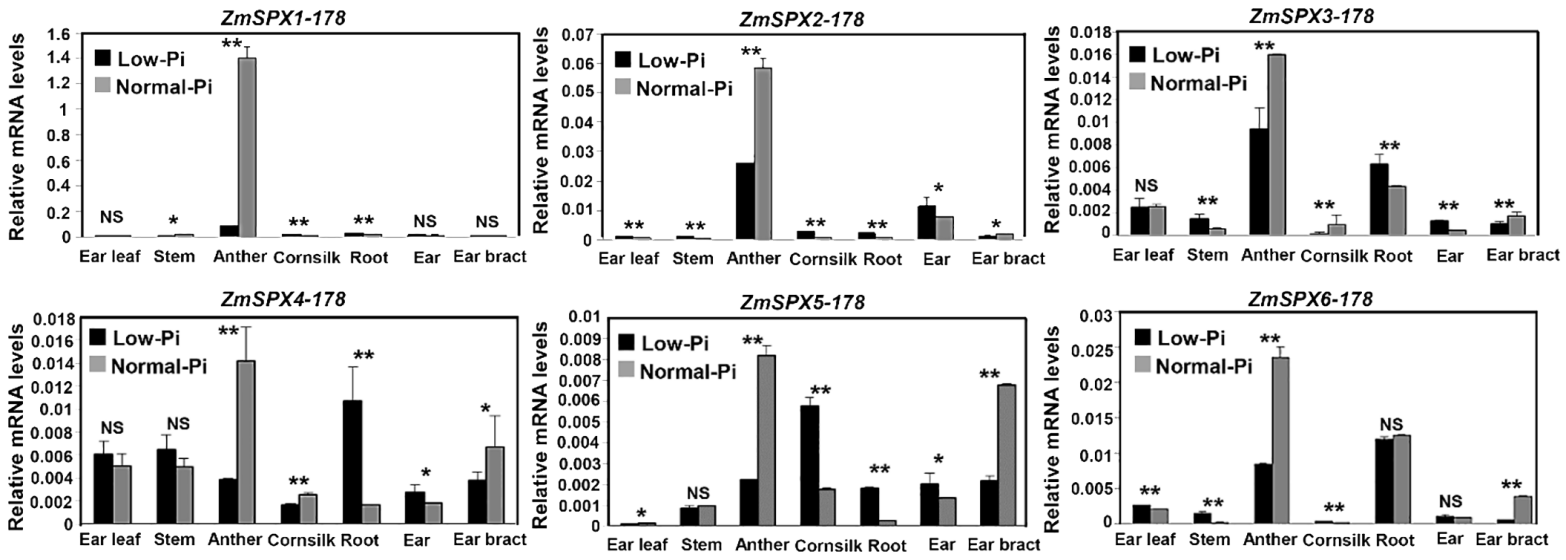
Expression pattern of ZmSPX family members in different tissues of 178 inbred lines under low- and normal-Pi conditions. Significant differences are indicated by Student’s *t*-test: *p < 0.05, **p < 0.01. NS, No significance.

Expression patterns of *ZmSPX*s were analyzed under low- and normal-Pi treatments in different tissues of 178 maize inbred lines, including ear leaf, stem, anther, cornsilk, root, ear, and ear bract (**Figure 2**). Notably, the *ZmSPX*s exhibited higher expression levels in anthers compared to other tissues but were significantly inhibited under low-Pi conditions. Meanwhile, *ZmSPX1*–*5* were induced by low-Pi stress in roots (**Figure 2**). Collectively, these results showed that the *ZmSPX* family members were involved in response to the low-Pi stress and play a specific function in different tissues of maize.

### 3.4 Subcellular localization of ZmSPX proteins

ZmSPX1, ZmSPX3, ZmSPX4, ZmSPX5, and ZmSPX6 were found to be localized in the nucleus and cytoplasm (**Supplementary Figure S3**). ZmSPX2 was mainly localized in the nucleus (**Supplementary Figure S3**). In a previous study (Xiao et al., 2021), subcellular localization studies of ZmSPXs in maize protoplasts revealed that ZmSPX1 (equivalent to ZmSPX5 in this study) and ZmSPX3 (equivalent to ZmSPX4 in this study) were localized in both the nucleus and cytoplasm. ZmSPX5 (equivalent to ZmSPX2 in this study) was exclusively localized in the nucleus. Moreover, ZmSPX4 (equivalent to ZmSPX6 in this study) and ZmSPX5 (equivalent to ZmSPX2 in this study) were detected in both the nucleus and cytoplasm, while ZmSPX6 (equivalent to ZmSPX1 in this study) was found in the chloroplast. The results showed partial consistency in the subcellular localization between tobacco tissue and maize protoplasts. However, some discrepancies were observed, presumably attributed to variations in cell types across different species.

### 3.5 Interaction between ZmSPXs and ZmPHRs in maize

**Figure 3:**
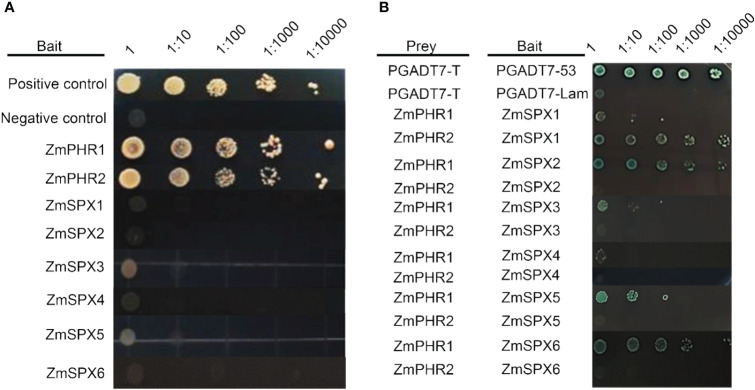
Yeast two-hybrid (Y2H) assay of ZmSPXs and ZmPHRs. **(A)** Self-activation analysis of ZmSPXs, ZmPHR1, and ZmPHR2. The medium is SD/−Leu-Trp-His-Ade (the medium lacking leucine, tryptophan, histidine, and adenine). **(B)** Y2H assay of ZmSPXs and ZmPHRs. PGADT7 and PGBKT7 vectors were used for positive and negative control, respectively. Yeast dilution ratio was 1, 1:10, 1:100, 1:1,000, and 1:10,000. The medium is SD/−Ade/−His/−Leu/−Trp/+X-α-Gal.

In this study, a yeast two-hybrid assay was conducted to identify the interaction between ZmSPX and ZmPHR proteins. Initially, the self-activation of *ZmSPX* and *ZmPHR* genes was assessed. Our findings showed that *ZmPHR1* and *ZmPHR2* demonstrated normal growth on SD/−Ade/−His/−Trp-deficient medium, while the *ZmSPX*s transformed with yeast cells did not grow on the selective medium (**Figure 3A**), indicating that the *ZmSPX*s had no self-activation effect. Therefore, SPX protein was chosen as bait and PHR protein as prey to verify the interaction. Furthermore, protein interaction was examined in various combinations of ZmSPXs, ZmPHR1, and ZmPHR2. Results revealed that combinations such as ZmSPX1 and ZmPHR2, ZmSPX2 and ZmPHR1, ZmSPX3 and ZmPHR1, ZmSPX5 and ZmPHR1, and ZmSPX6 and ZmPHR1 all exhibited robust growth on SD/−Ade/−His/−Leu/−Trp/+X-α-gal medium, indicating their protein interactions (**Figure 3B**). It is suggested that *ZmSPX*s and *ZmPHR*s may modulate the maize response to low-Pi stress at the post-transcriptional level.

### 3.6 Overexpressing and knocking out *ZmSPX1* in maize revealed its correlation with Pi absorption and its effect on yield and yield-related traits

**Figure 4:**
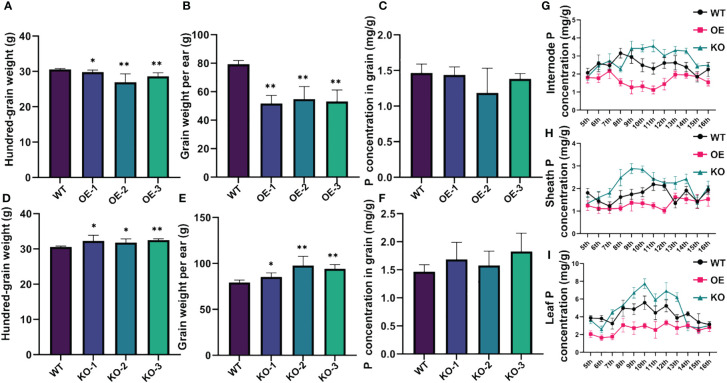
Phenotypic identification of *ZmSPX1* transgenic maize materials. **(A–C)** Statistical analysis of differences in hundred-grain weight, grain weight per ear, and P concentration in grain between *ZmSPX1* overexpressing lines and wild-type lines. **(D**–**F)** Statistical analysis of differences in hundred-grain weight, grain weight per ear, and P concentration in grain between *ZmSPX1* knockout lines and wild-type lines. **(G**–**I)** The internode P concentration, sheath P concentration, and leaf P concentration in *ZmSPX1* overexpressing lines, knockout lines, and wild-type lines. Significant differences are indicated by Student’s *t*-test: *p < 0.05, **p < 0.01.

To investigate the function of *ZmSPX*s in response to low-Pi stress, overexpression lines of *ZmSPX*s were created in *Arabidopsis*. However, only the overexpression of *ZmSPX1* served to enhance root sensitivity to Pi deficiency and high-Pi conditions in *A. thaliana* (**Supplementary Figures S4**, **S5**). In order to further explore the function and role of *ZmSPX1*, we generated knockout and overexpressing maize transgenic lines for this gene. Our experimental results demonstrated a significant reduction in the hundred-grain weight and grain weight per year in the overexpressing lines compared to the wild type, while the knockout lines exhibited a marked increase (**Figures 4A, B, D, E**). Moreover, analysis of the P concentration in the grains of the knockout and overexpressing lines revealed no significant difference compared to the wild type (**Figures 4C, F**). We also assessed the P concentration in the internode, sheath, and leaf of the transgenic plants in the field trials. The findings indicated a higher P concentration in the knockout lines in these tissues than in the wild-type lines, whereas the overexpressing lines showed lower P concentrations compared to the wild-type lines (**Figures 4G–I**). These results suggested that *ZmSPX1* affected the P concentration in maize stems and leaves and exerted a certain impact on the yield of maize, but not on the grains.

### 3.7 Sequence variation and association analysis of *ZmSPX1*

To identify significant variations associated with phenotypic traits, we performed genome sequence amplification on 211 inbred lines and conducted multiple sequence alignments. We obtained a total of 1,390 bp of sequences, comprising 780 bp upstream of the initiation codon, 354 bp of the coding region, and 256 bp downstream of the initiation codon (**Supplementary Table S10**). We identified a total of 41 variants, which included 34 SNPs and seven InDels. The average distances between SNPs and InDels were 40.89 bp and 198.57 bp, respectively. The sequence variation frequencies varied across regions, with the highest frequency observed in the upstream sequence of the initiation codon (0.038) and the lowest in the coding region (0.006) (**Supplementary Table S10**). By utilizing a 200-bp sliding window with a 50-bp step size, we analyzed the nucleotide diversity (π × 1,000) of the *ZmSPX1*. We observed the overall nucleotide diversity to be 0.009, with the upstream sequence of the initiation codon exhibiting the highest diversity (0.013) among all regions and the coding region displaying the lowest diversity (0.002). Additionally, we investigated the selection pressure of *ZmSPX1* using Tajima’s D, Fu and Li’s D*, and F* tests (**Supplementary Table S10**). The results showed that the upstream sequences displayed positive values, indicating a mode of balanced selection in their sequence evolution, with both Tajima’s D and Fu and Li’s F* tests being significant (**Supplementary Table S10**). Conversely, the coding region and downstream showed negative values, suggesting that these regions have experienced either negative selection or population expansion (**Supplementary Table S10**).

**Figure 5:**
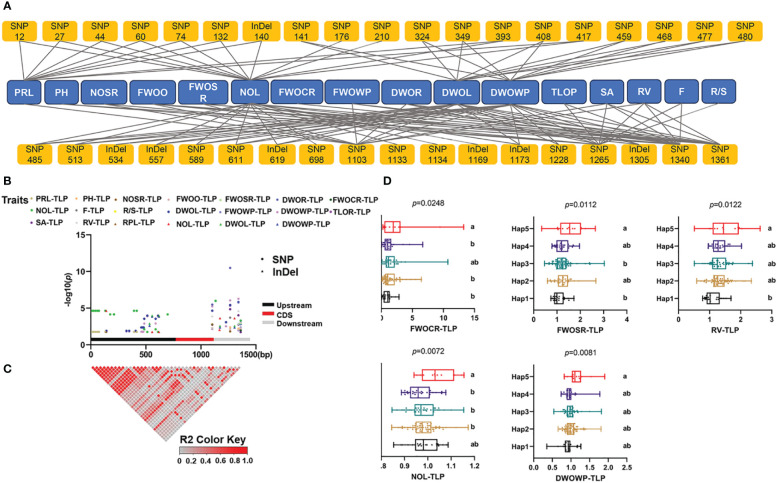
Candidate gene association analysis of *ZmSPX1* with traits of low-Pi tolerance index. **(A, B)** The results of the candidate gene association analysis. **(C)** Linkage disequilibrium (LD) heatmap of *ZmSPX1*. **(D)** Phenotypic difference between different haplotypes. The abbreviation for each trait: primary root length (PRL), plant height (PH), number of seminal roots (NOSR), fresh weight of overground (FWOO), fresh weight of seminal root (FWOSR), dry weight of root (DWOR), fresh weight of crown root (FWOCR), number of leaves (NOL), number of root forks **(F)**, root/shoot ratio (R/S), dry weight of leaves (DWOL), fresh weight of whole plant (FWOWP), dry weight of whole plant (DWOWP), total length of root (TLOR), root surface area (SA), and root volume (RV). TLP means the low-Pi tolerance index (Low-Pi/Normal-Pi). Means with the same letter in **(D)** are not significantly different at *p* < 0.05 according to one-way ANOVA followed by Tukey’s multiple comparison test.

Additionally, to further investigate the relationship between sequence variations of *ZmSPX1* and the phenotype in the maize seedling stage, we employed an MLM for candidate gene association analysis. We identified, under normal-Pi treatment, 37 markers (30 SNPs and seven InDels) as significantly associated with 17 traits, explaining 2.6% to 8.6% of the phenotypic variation (**Supplementary Table S11**). We found, under low-Pi treatment, a total of 10 markers to be significantly correlated with 13 traits, with *R* ^2^ ranging from 2.8% to 5.4% (**Supplementary Table S12**). Moreover, 37 markers were significantly correlated with 16 traits of low-Pi tolerance index, explaining 2.8% to 20.2% of the phenotypic variation (**Figures 5A, B**; **Supplementary Table S13**). As the low-Pi tolerance index integrated traits under low- and normal-Pi conditions, we utilized all sequence variant sites significantly associated with the low-Pi tolerance index for haplotype division. First, LD analysis revealed the presence of multiple LD blocks with high linkage relationships (**Figure 5C**). Then, we categorized all variant sites into five haplotypes (MAF > 0.05) and conducted comparisons among the different haplotypes (**Figure 5D**; **Supplementary Table S14**). The findings indicated that in the low-Pi tolerance index of root traits, the fresh weight of the crown root index of Hap5 was significantly higher than that of Hap1, Hap2, and Hap4, while the fresh weight of the seminal root index was higher than that of Hap3 and Hap1. Additionally, the root volume of Hap5 was significantly higher than that of Hap1. Furthermore, we observed that the dry weight of the whole plant index of Hap5 was significantly higher than that of other haplotypes. Based on these results, it can be inferred that Hap5 enhances biomass production by promoting root development.

## 4 Discussion

### 4.1 The maize transgenic lines of *ZmSPX1* exhibited significant impacts on P concentration and yield

Pi homeostasis is crucial for plant growth and development, with *ZmSPX1* playing a pivotal role in Pi uptake by plants from both the source and reservoir. Consequently, we first analyzed the phenotypes in *ZmSPX1* overexpression transgenic *A. thaliana* and WT under different Pi concentration treatments. Under both Pi deficiency and high-Pi conditions, we observed a more robust root system development in the WT plants, whereas the growth of the root system was inhibited in the overexpressing (OE) lines. These results indicated that overexpression of the *ZmSPX1* gene in *A. thaliana* enhanced the sensitivity of roots to both Pi deficiency and high-Pi conditions. Therefore, we proceeded to construct transgenetic lines for *ZmSPX1* in maize. The knockout *ZmSPX1* in maize led to an increase in P content in the stems and leaves. Additionally, a significant rise in hundred-grain weight and grain yield per ear was also observed in the knockout maize lines. Conversely, overexpression of *ZmSPX1* in maize demonstrated contrasting phenotypes. Studies in dicotyledonous model plants have demonstrated that *SPX*s, functioning as negative regulators, can impede the central regulator *PHR1* from acting on downstream low-Pi response genes (Jia et al., 2023). Conversely, research reports in monocotyledonous model plants such as *A. thaliana* and soybean suggest that *SPX*s serve as positive regulators. Overexpression of *SPX1* induced the expression of low-Pi response genes and thus increased plant P concentration (Duan et al., 2008; Yao et al., 2014a). These results indicated that the expression patterns and functions of the *SPX1* gene in maize and *A. thaliana* are different, which may also explain why the P concentration in *A. thaliana* overexpressing lines of *SPX* was higher than that in wild-type plants under normal-Pi conditions.

Additionally, a previous study demonstrated that *OsPHO1;2* and *ZmPHO1;2* play a crucial role in Pi allocation during grain filling. The activity of ADP-glucose pyrophosphorylase (AGPase) was inhibited in the knockout mutants of *OsPHO1;2* and *ZmPHO1;2*, resulting in a defect in grain filling and a reduction in hundred-grain weight (Ma et al., 2021). Regarding the plant Pi signaling pathway (Wang et al., 2021b), the E2 ubiquitin-binding enzyme PHO2 can degrade PHO1 through its N-terminal SPX domain. The mRNA level of *PHO2* was regulated by the cleavage of miR399. PHR1 can bind to the promoter region of miRNA399 to modulate its expression. Therefore, the relationship between SPX proteins and PHO1 was established through this network. However, further research is needed to investigate whether the impact of *ZmSPX1* on maize grain yield, as observed in this study, is related to *ZmPHO1*.

### 4.2 Association analysis of the *ZmSPX1* gene: implications for low-Pi signaling pathway and marker development for efficient Pi utilization in maize

To further investigate the relationship between the *ZmSPX1* gene and low-Pi stress response-related traits, we conducted an association analysis utilizing sequence variations of the gene in the population and integrated the corresponding phenotypes. The results revealed that variant sites in *ZmSPX1* are significantly associated with numerous low-Pi-related traits. This finding complements the findings from the phenotypic identification experiments in *Arabidopsis* involving the overexpression of the *ZmSPX1* gene, providing strong evidence for the pivotal role of the *ZmSPX1* gene in the low-Pi stress response pathway in maize. Additionally, we identified beneficial haplotypes based on 37 markers significantly associated with low-Pi tolerance indexes. Among these, Hap5 manifested a notable low-Pi tolerance phenotype, presenting an opportunity for the development of low-Pi-tolerant maize materials based on this outcome, and the identification of novel markers for screening purposes.

## 5 Conclusion

In conclusion, our study delineated the evolutionary relationship of maize *SPX*s with counterparts in other plants, particularly revealing a close relationship and collinearity with rice *SPX* genes. The presence of hormone-responsive elements in *ZmSPX*s promoters suggests their involvement at the nexus of hormone and Pi signaling. Expression pattern analyses indicated that *ZmSPX*s genes were upregulated under low-Pi stress, with pronounced expression in anthers and roots, and were localized to the nucleus and cytoplasm. The interaction of ZmSPXs with PHR proteins underscored the significance of *SPX*s in maize’s low-Pi stress signaling pathways. Specifically, overexpression of *ZmSPX1* can enhance root sensitivity to Pi deficiency and high-Pi conditions in *A. thaliana*, emphasizing the pivotal role of the *SPX* gene family in the Pi stress response in maize. Finally, *ZmSPX1* transgenic maize lines exhibited changes in P concentration in different tissues and yield, demonstrating that *ZmSPX1* plays an important role in regulating the transport and distribution of P in maize and ultimately influencing yield. These findings will provide valuable information for further investigations into the mechanisms of the *SPX*-mediated Pi signaling pathway.

## Data availability statement

The datasets presented in this study can be found in online repositories. The names of the repository/repositories and accession number(s) can be found in the article/**Supplementary Material**.

## Author contributions

BL: Conceptualization, Formal analysis, Funding acquisition, Methodology, Visualization, Writing – original draft, Writing – review & editing. JS: Conceptualization, Formal analysis, Methodology, Validation, Visualization, Writing – original draft, Writing – review & editing. HZ: Formal analysis, Visualization, Writing – original draft. JZ: Writing – review & editing. GY: Writing – review & editing. WW: Writing – review & editing. JG: Writing – review & editing. SZ: Writing – review & editing. PM: Writing – review & editing. ZN: Writing – review & editing. XZ: Writing – review & editing. DL: Project administration, Resources, Writing – review & editing. LW: Project administration, Resources, Writing – review & editing. DG: Project administration, Resources, Writing – review & editing. SQG: Project administration, Resources, Writing – review & editing. SS: Project administration, Resources, Writing – review & editing. ZG: Investigation, Writing – review & editing. SBG: Writing – review & editing, Conceptualization, Funding acquisition, Methodology, Project administration.

## Supplementary material

Supplementary Figure 1 CRISPR/Cas9-induced mutation in the *ZmSPX1* gene.

Supplementary Figure 2 Detection of expression level of *ZmSPX1* gene in overexpressing plants. Significant differences are indicated by Student’s *t*-test: **P* < 0.05, ***P* < 0.01.

Supplementary Figure 3 Subcellular localization of ZmSPXs in tobacco epidermal. GFP, green fluorescence protein.

Supplementary Figure 4 Detection of overexpression of the *ZmSPX1* in *Arabidopsis thaliana*. M: DNA marker BM2000.

Supplementary Figure 5 Characterization of Arabidopsis thaliana ZmSPX1-overexpression plants in response to low-Pi conditions. **(A)** The WT and OE seedlings were subjected to treatments with P concentrations of 0 mM, 0.1 mM, 1 mM, and 10 mM for 14 days. **(B-C)** The expression of AtPHR1 and ZmSPX1 in WT and OE. The numbers 1 and 2 on the abscissa represent different independent replicates. **(D-F)** Measurement of root traits. Significant differences are indicated by Student’s t-test: *P < 0.05, **P < 0.01. Under Pi-free and high-Pi conditions, only the *ZmSPX1* overexpression lines exhibited negative regulation of root growth when compared with the WT. Phenotypic of OE and WT was analyzed under Pi deficiency (0 mmol/L), low-Pi (0.1 mmol/L), normal-Pi (1 mmol/L) and high-Pi (10 mmol/L) treatments. The results indicated that, except under low-Pi conditions, the total root length, total root projected area, and total root surface area of the OE were significantly lower than those of the WT. Furthermore, the expression patterns of the *AtPHR1* and *ZmSPX1* genes were analyzed in the WT and OE plants. The results demonstrated significant downregulation of the *AtPHR1* in the OE plants and substantial upregulation in the WT plants. In contrast, *ZmSPX1* was upregulated in the OE plants and downregulated in the WT plants. Concurrently, we also examined the nitrogen and P concentration of the OE and WT (**Supplementary Figure S6**). Under low-Pi conditions, the nitrogen concentration in the OE was notably lower than that in the WT. However, under 1mM Pi concentration treatment, the nitrogen concentration in the OE was significantly higher than in the WT, while at 10mM Pi concentration conditions, the difference in nitrogen concentration between OE and WT was not statistically significant. Under Pi deficiency and low-Pi conditions, there was no significant difference in P concentration between the OE and WT. Nevertheless, at Pi concentrations of 1mM and 10mM, the P concentration in the OE was markedly elevated compared to that in the WT. Owing to the exceptionally low biomass of Arabidopsis tissues, there was a potential for deviations in the measurement of nutrient concentrations, and no consistent results were observed with regard to root traits. As a result, this study advanced to develop overexpression and knockout lines for *ZmSPX1* in maize to investigate the gene’s impact on P concentration in maize tissues.

Supplementary Figure 6 The nitrogen and P concentrations of ZmSPX1 overexpression Arabidopsis lines under varying Pi levels. **(A)** Nitrogen concentrations. **(B)** P concentrations.
