# Supplementary material for: SPX family response to low phosphorus stress and the involvement of ZmSPX1 in phosphorus homeostasis in maize
Source: Front Plant Sci. 2024 Jul 8;15:1385977. doi: 10.3389/fpls.2024.1385977 (PMC11260721; doi:10.3389/fpls.2024.1385977)
Supplement: Supplementary Figure 1 — CRISPR/Cas9-induced mutation in the ZmSPX1 gene. [file DataSheet_1.pdf]

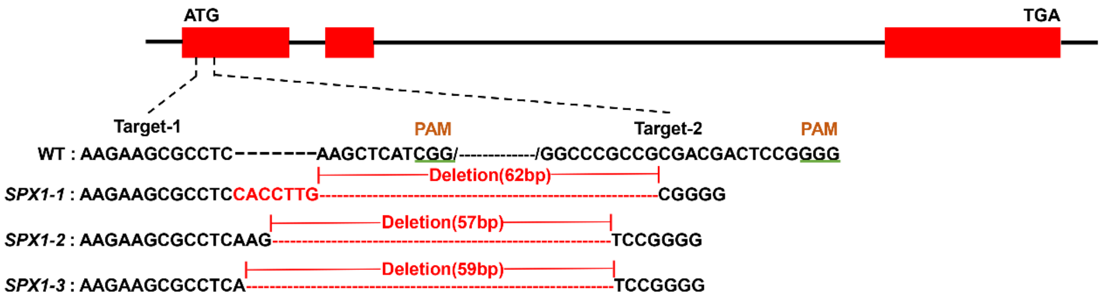

Figure S1. CRISPR/Cas9-induced mutation in the *ZmSPX1* gene.

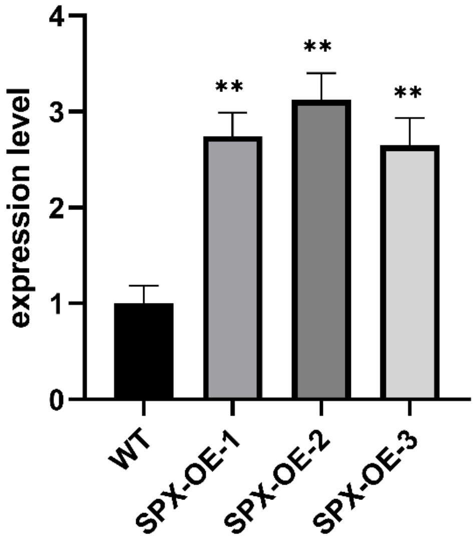

Figure S2. Detection of expression level of *ZmSPX1* gene in overexpressing plants. Significant differences are indicated by Student's *t*-test: *P < 0.05, **P < 0.01.

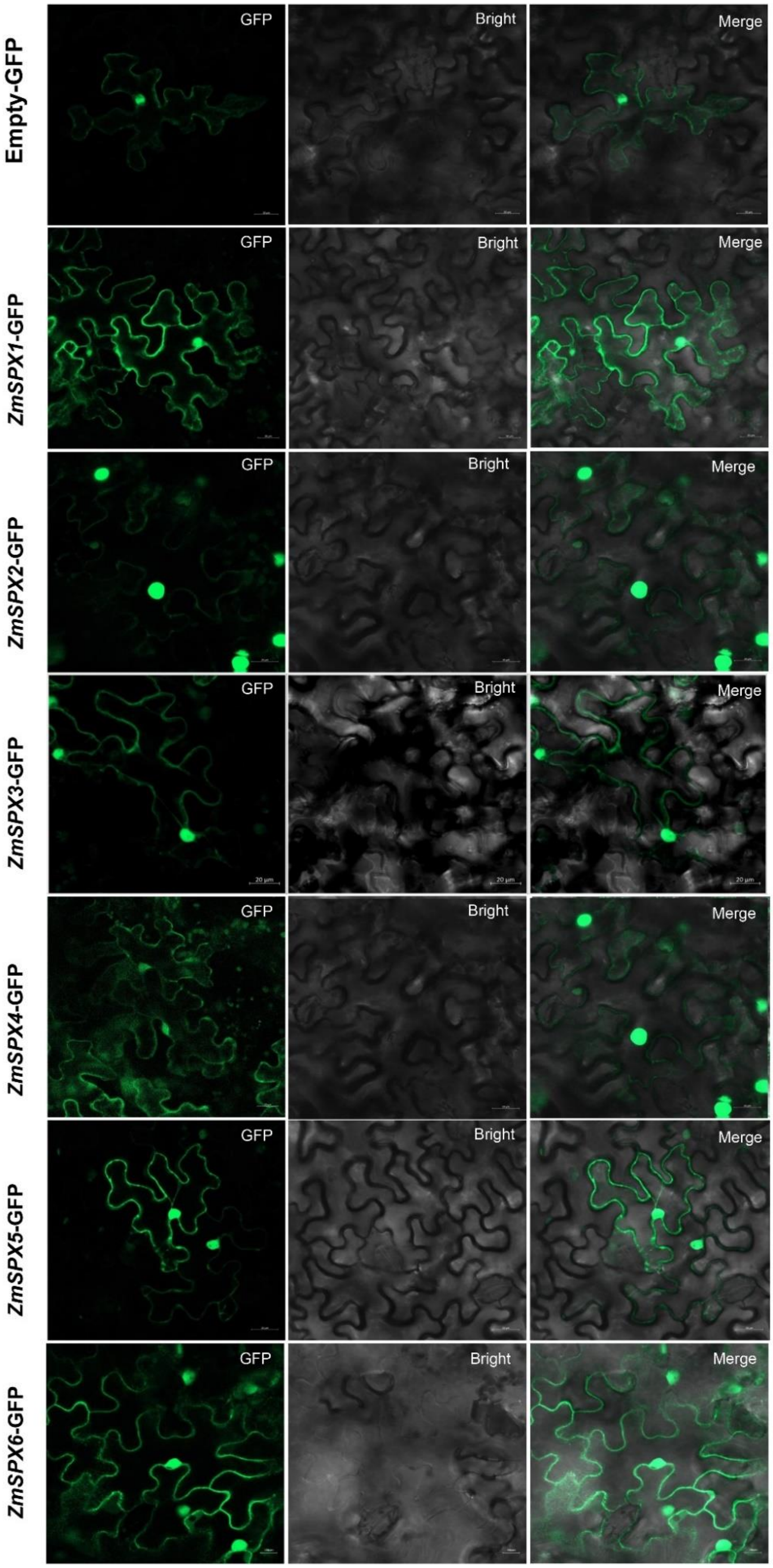

Figure S3. Subcellular localization of ZmSPXs in tobacco epidermal. GFP, green fluorescence protein.

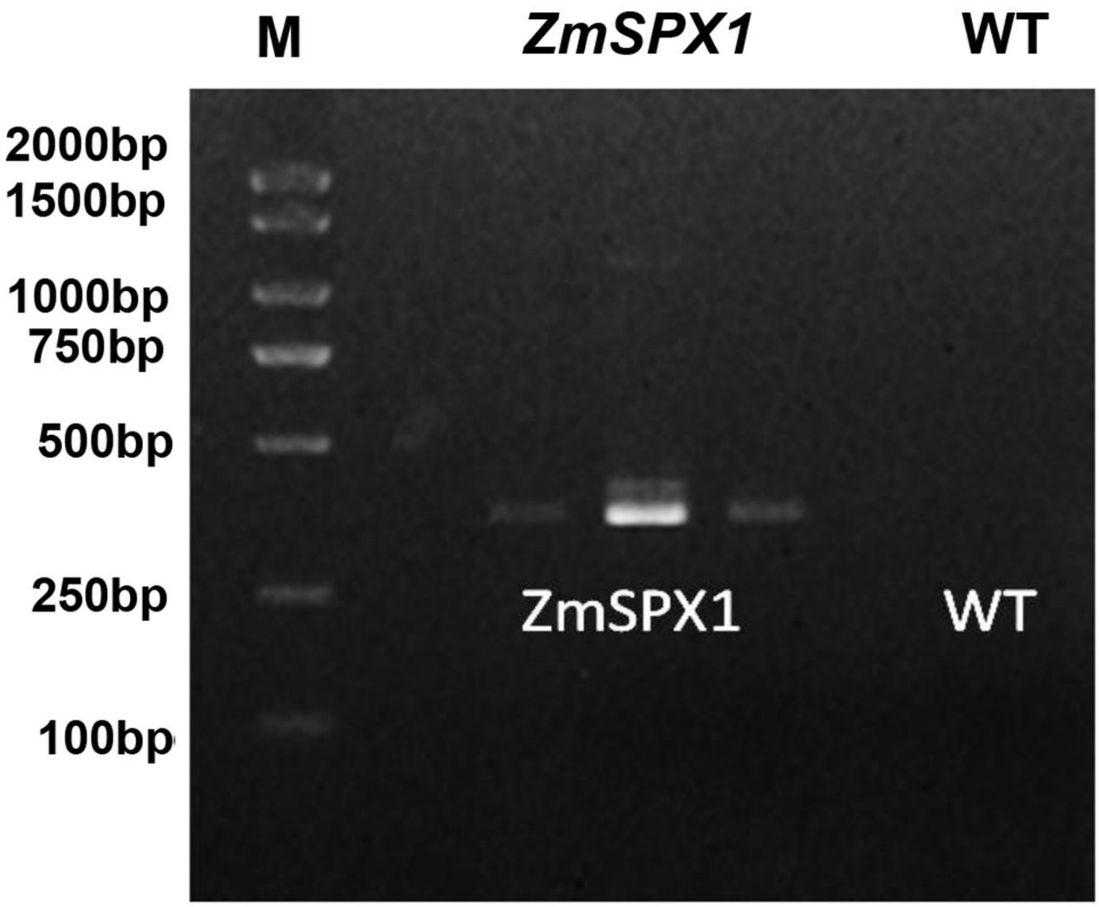

Figure S4. Detection of overexpression of the *ZmSPX1* in *Arabidopsis thaliana*; M: DNA marker BM2000.

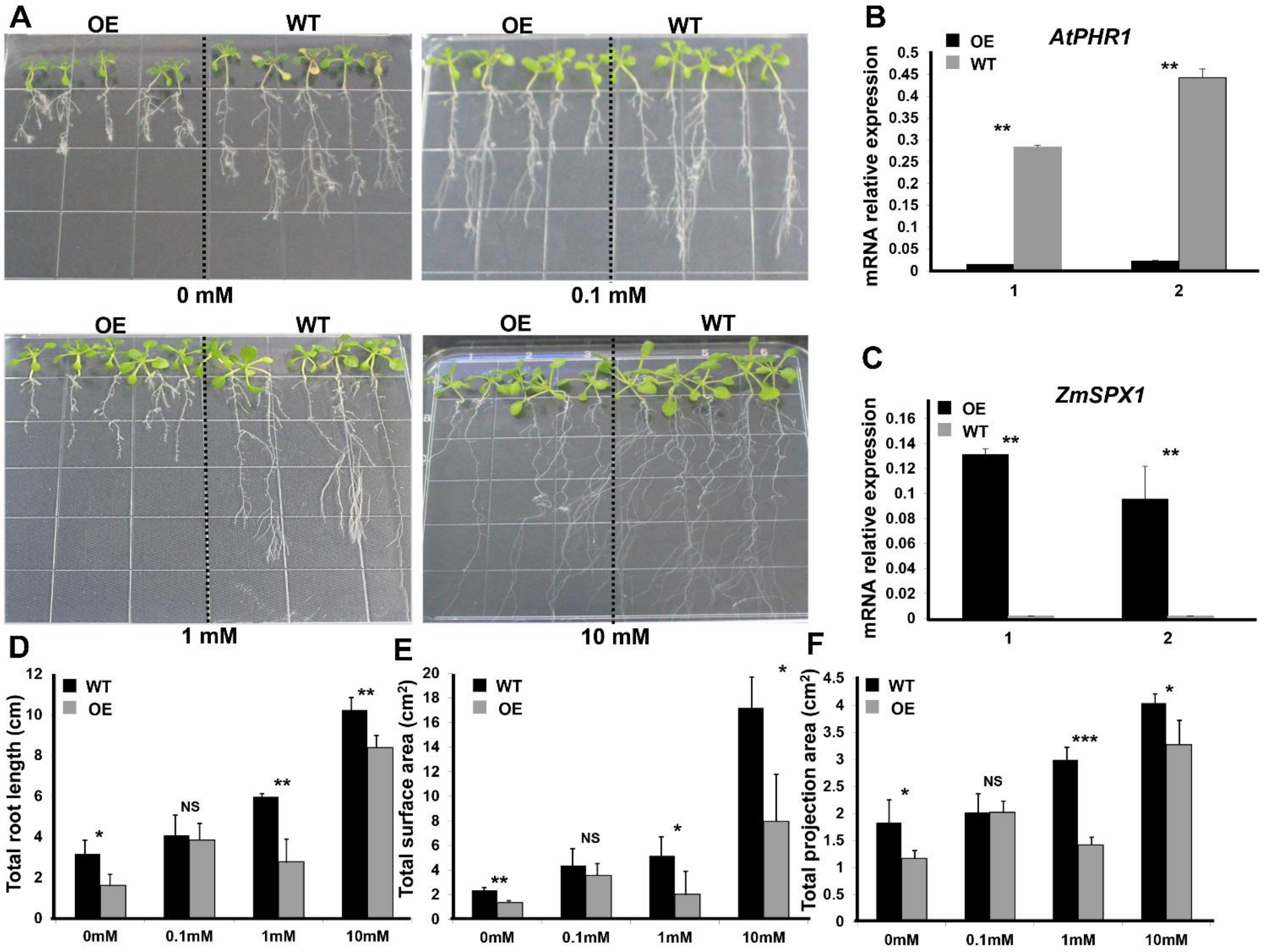

Figure S5. Characterization of Arabidopsis thaliana ZmSPX1-overexpression plants in response to low-Pi conditions. (A) The WT and OE seedlings were subjected to treatments with P concentrations of 0 mM, 0.1 mM, 1 mM, and 10 mM for 14 days. (B-C) The expression of AtPHR1 and ZmSPX1 in WT and OE. The numbers 1 and 2 on the abscissa represent different independent replicates. (D-F) Measurement of root traits. Significant differences are indicated by Student's t-test: *P < 0.05, **P < 0.01.

Note: Under Pi-free and high-Pi conditions, only the *ZmSPX1* overexpression lines exhibited negative regulation of root growth when compared with the WT. Phenotypic of OE and WT was analyzed under Pi deficiency (0 mmol / L), low-Pi (0.1 mmol / L), normal-Pi (1 mmol / L) and high-Pi (10 mmol / L) treatments. The results indicated that, except under low-Pi conditions, the total root length, total root projected area, and total root surface area of the OE were significantly lower than those of the WT. Furthermore, the expression patterns of the *AtPHR1* and *ZmSPX1* genes were analyzed in the WT and OE plants. The results demonstrated significant downregulation of the *AtPHR1* in the OE plants and substantial upregulation in the WT plants. In contrast, *ZmSPX1* was upregulated in the OE plants and downregulated in the WT plants. Concurrently, we also examined the nitrogen and P concentration of the OE and WT (Figure S6). Under low-Pi conditions, the nitrogen concentration in the OE was notably lower than that in the WT. However, under 1mM Pi concentration treatment, the nitrogen concentration in the OE was significantly higher than in the WT, while at 10mM Pi concentration conditions, the difference in nitrogen concentration between OE and WT was not statistically significant. Under Pi deficiency and low-Pi conditions, there was no significant difference in P concentration between the OE

and WT. Nevertheless, at Pi concentrations of 1mM and 10mM, the P concentration in the OE was markedly elevated compared to that in the WT. Owing to the exceptionally low biomass of Arabidopsis tissues, there was a potential for deviations in the measurement of nutrient concentrations, and no consistent results were observed with regard to root traits. As a result, this study advanced to develop overexpression and knockout lines for *ZmSPX1* in maize to investigate the gene's impact on P concentration in maize tissues.

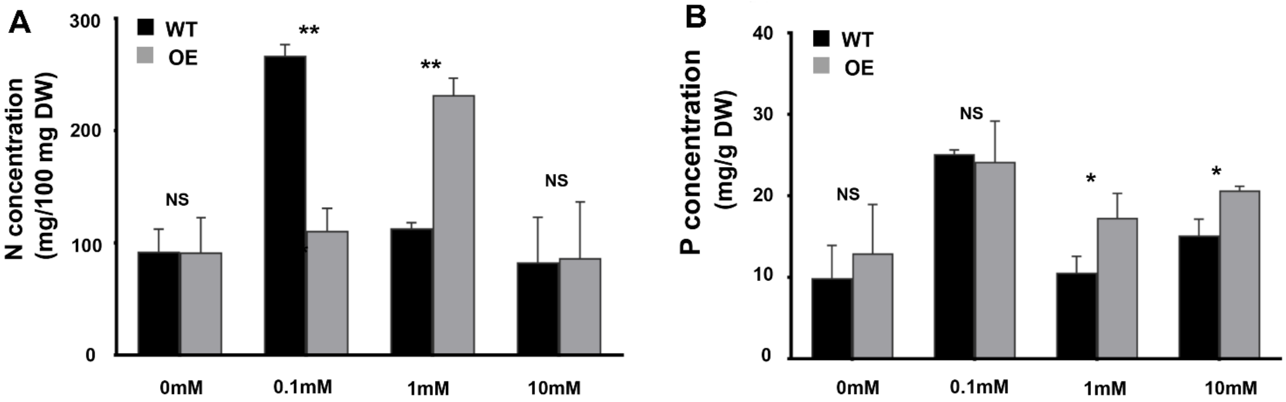

Figure S6. The nitrogen and P concentrations of *ZmSPX1* overexpression Arabidopsis lines under varying Pi levels. **(A)** Nitrogen concentrations. **(B)** P concentrations.
